# Does the Chemodiversity of Bacterial Exometabolomes Sustain the Chemodiversity of Marine Dissolved Organic Matter?

**DOI:** 10.3389/fmicb.2019.00215

**Published:** 2019-02-14

**Authors:** Beatriz E. Noriega-Ortega, Gerrit Wienhausen, Andrea Mentges, Thorsten Dittmar, Meinhard Simon, Jutta Niggemann

**Affiliations:** ^1^Institute for Chemistry and Biology of the Marine Environment, University of Oldenburg, Oldenburg, Germany; ^2^Leibniz-Institute of Freshwater Ecology and Inland Fisheries, Berlin, Germany; ^3^Helmhotz Institute for Functional Marine Biodiversity (HIMFB), University of Oldenburg, Oldenburg, Germany

**Keywords:** chemodiversity, Roseobacter, DOM, exometabolomes, marine heterotrophic bacteria

## Abstract

Marine dissolved organic matter (DOM) is a complex mixture of chemical compounds. At 750 Pg C, it is one of the biggest pools of reduced carbon on Earth. It has been proposed that the diversity of DOM is responsible for its recalcitrance. We hypothesize that the chemodiversity of marine DOM is a reflection of the chemodiversity of bacterial exometabolomes. To test this, we incubated two model strains of the *Roseobacter* group; *Phaeobacter inhibens* and *Dinoroseobacter shibae* in pure culture using three different simple organic compounds as sole carbon sources (glutamate, glucose, and acetate and succinate for *P. inhibens* and *D. shibae*, respectively). The exometabolome of the model organisms was characterized using Fourier Transform Ion Cyclotron Resonance Mass Spectrometry (FT-ICR-MS) and ecological diversity measures. We detected thousands of molecular masses in the exometabolomes of *P. inhibens* and *D. shibae* (21,105 and 9,386, respectively), reflecting the capability of single bacterial strains to diversify simple organic compounds. The chemical composition of the exometabolomes changed with growth phase and also differed according to the strain incubated and the utilized substrate. We mimicked a higher diversity of substrates, bacterial species and heterogeneous growth (different growth phases) to approach the complexity of natural environments, by computationally creating combinations of detected exometabolomes. We compared the chemodiversity of these combinations, indicative for chemodiversity of freshly produced microbial DOM to that of refractory DOM from one of the oldest oceanic water masses (North Equatorial Pacific Intermediate Water). Some combinations of exometabolomes showed higher richness than the deep ocean refractory DOM, and all the combinations showed higher functional diversity. About 15% of the 13,509 molecular formulae detected in exometabolomes and refractory oceanic DOM were shared, i.e., occurred in *Roseobacter* exometabolomes and in deep water samples. This overlap provides further support for our hypothesis that marine bacteria from the *Roseobacter* group contribute to the sustainability of marine DOM chemodiversity and stability.

## Introduction

Marine dissolved organic matter (DOM) is one of the most complex molecular mixtures on our planet. Thousands (>10,000) of compounds have been identified within this mixture (Riedel and Dittmar, 2014). Major sources of marine DOM are primary production by phytoplankton and subsequent release processes (Azam and Malfatti, 2007; Kujawinski, 2011). DOM is operationally classified into reactivity fractions ranging from labile to ultra-refractory (Hansell, 2013). Most of the freshly produced DOM is labile and turned over within seconds to hours; hence the size of the labile DOM pool is small. The refractory fraction, in contrast, accumulates in the ocean for decades to millennia (Williams et al., 1969; Bauer et al., 1992; Hansell, 2013) and comprises 70 – 95% of total DOM (Hedges et al., 2000; Ogawa et al., 2001). The reasons for the long-term stability of DOM in the oceans are still discussed (Dittmar, 2015). The “dilution hypothesis” proposes that given the extremely high chemodiversity of DOM, the concentrations of single compounds are very low thus limiting the encounter rate of microbes to identical molecules and preventing microbial degradation (Barber, 1968; Arrieta et al., 2015).

The processes leading to the diversification of DOM have not been fully identified, but previous studies indicate that prokaryotic microorganisms play a key role. It has been shown that freshly produced organic matter is transformed during subsequent microbial degradation into a complex mixture of compounds that potentially contributes to refractory DOM, consistent with the concept of the microbial carbon pump (Ogawa et al., 2001; Jiao et al., 2010; Kujawinski, 2011; Koch et al., 2014; Osterholz et al., 2015). In fact, recent studies revealed diversification of simple organic molecules and freshly produced DOM by bacterial communities using molecular characterization via FT-ICR-MS (Koch et al., 2014; Lechtenfeld et al., 2015; Osterholz et al., 2015).

A large fraction of DOM molecules falls in the mass range of 250–550 Da (Aiken and Malcolm, 1987; Simpson, 2002), which is typical for microbial metabolites (Carlson et al., 2007) and consistent with a scenario where DOM is mainly composed of microbial metabolites (Dittmar and Stubbins, 2014). Metabolites originate from cellular activity and are mostly low molecular weight compounds (<1500 Da; Oliver et al., 1998). The set of metabolites at a specific physiological condition is termed the metabolome; it reflects the final response of cells to certain environmental conditions. The endo-metabolome includes all intracellular metabolites, whereas the exometabolome includes only those that are released to the environment (Allen et al., 2003; Mapelli et al., 2008). Many different compounds are produced and released by marine bacteria even when growing on simple organic carbon sources (Rosselló-Mora et al., 2008; Romano et al., 2014; Fiore et al., 2015), or in defined co-culture with selected phytoplankton (Kujawinski et al., 2009). The exact identity of most of these compounds is unknown, however, it has been inferred that some are intermediates in biosynthetic pathways for compounds like vitamins and growth factors, suggesting these exometabolomes act as market places of microbial metabolites (Romano et al., 2014; Fiore et al., 2015; Mas et al., 2016; Wienhausen et al., 2017).

Diversity is a property of communities that can be assessed by a variety of statistical indices. In analogy to classical ecological concepts, molecular mixtures (e.g., plant extracts, human secretions, marine DOM, and bacterial exometabolomes) can be considered as communities of species that are detected in different relative abundances. In this study, we apply ecological indices to quantitatively assess the chemical diversity (chemodiversity) of molecular mixtures. Universally accepted units for diversity do not exist, which makes the analysis of this property challenging (Shade, 2017). Implementation of appropriate measures for DOM chemodiversity is instrumental for the comparison among different molecular mixtures which poses a crucial step in constraining sources and fate of marine DOM (Landa et al., 2014). DOM chemodiversity can be looked at from different angles: by either counting detected compounds (molecular richness), describing the abundance distribution of detected molecular masses (abundance-based diversity), or characterizing the variability of their chemical properties (functional diversity; Tilman, 2001). All these measures capture complementary aspects of DOM chemodiversity and should be applied jointly: Through an entire latitudinal transect along the Atlantic ocean, the number of molecular formulae (MF) in marine DOM was relatively constant, while the functional diversity decreased with increasing degradation state of DOM (Mentges et al., 2017).

As a consequence of these considerations, we hypothesize that (a) the exometabolome of single bacterial strains is molecularly similarly diverse as natural marine DOM and (b) the chemodiversity of exometabolomes further increases when different growth conditions and strains are considered. Ultrahigh resolution mass spectrometry (FT-ICR-MS) yields thousands of molecular formulae (MF) per sample, including compounds that would remain uncharacterized using conventional analytical techniques. We applied FT-ICR-MS to unravel exometabolome composition and chemodiversity of ecologically relevant bacteria from the *Roseobacter* group. Members of the *Roseobacter* group are known to be abundant and highly active during algal blooms (Teeling et al., 2012; Buchan et al., 2014; Segev et al., 2016), suggesting they play an important role in organic matter transformation. We used two well characterized model organisms of the *Roseobacter* group, *Phaeobacter inhibens* and *Dinoroseobacter shibae*, and characterized the composition and chemodiversity of their exometabolomes. The results reported here are based on the same data as the study presented by Wienhausen et al. (2017), who report the detection of specific precursors of vitamins, amino acids, growth factors and quorum sensing molecules in the exometabolome, suggesting that the investigated strains act as helpers for other marine microbes by providing biosynthetic precursors and other molecules as public goods. Here, we focus on the entirety of exometabolites to unravel the metabolic potential of bacteria from the *Roseobacter* group in terms of molecular composition and diversity. We include an *in silico* approach to test whether marine DOM can be explained as a reflection of the sum of multiple marine bacterial exometabolomes.

## Materials and Methods

### Bacterial Strains

*Dinoroseobacter shibae* DSM 16493 (GenBank: CP000830.1) and *Phaeobacter inhibens* DSM 17395 (GenBank: CP002976.1) are representative members of the *Roseobacter* group. Both organisms are fully genome-sequenced (Wagner-Döbler et al., 2010; Thole et al., 2012) with genome sizes of 4,417,868 and 4,227,134 base pairs (Kyoto Encyclopedia of Genes and Genomes KEGG) database (Kanehisa and Goto, 2000), including 4,194 and 3,875 protein encoding genes and 50 and 69 RNA genes for *D. shibae* and *P. inhibens*, respectively.

### Incubations and Sampling

The experiments were performed as described in Wienhausen et al. (2017). All materials were acid washed (pH 2, ultrapure water) and all glassware was additionally combusted at 500°C for 3 h in order to prevent potential contaminations with organic compounds that could be detected by our high sensitivity analytics and bias analyses outcome and data interpretation. All chemicals used were from analytical grade or higher.

Artificial seawater (ASW) was prepared as described by Zech et al. (2009). For *P. inhibens*, non-chelated trace elements were added. The medium for *D. shibae* included chelated trace element solution and vitamins. *P. inhibens* is able to produce chelators itself which allows these bacteria to grow on the medium with non-chelated trace elements. *D. shibae* on the other hand needs the exogenous chelating agents.

To the ASW medium, 20 mM of glutamic acid, 35 mM of acetate and 4 mM of glucose were added for *P. inhibens*. For *D. shibae*, 8 mM of glutamic acid, 10 mM of succinate and 5 mM of glucose were used. *D. shibae* did not reach the desired optical density when growing on acetate, therefore succinate was used as an alternative acid. Different concentrations of carbon were used as substrates in order to achieve similar growth yields among the different incubations.

Bacteria were transferred from a glycerol stock to marine broth medium (MB) and grown to exponential growth phase. To reduce the carry-over metabolism, bacteria were repeatedly cultivated in ASW with the one single organic carbon source used for the respective incubation and transferred five times when they had reached exponential phase. Additionally, to reduce the medium carry over, cell pellets were washed three times with ASW before inoculation of the final experiment flasks. Both bacterial strains were incubated in 500 ml of medium in 2 l baffled Erlenmeyer flasks at 28°C (Biebl et al., 2005; Martens et al., 2006) in the dark, shaking at 100 rpm. Triplicates were incubated and sampled in parallel, as well as a sterile control for each substrate (sterility verified by optical density and flow cytometry).

Samples for DOC and substrate quantification and for DOM characterization were taken at the beginning of the incubation. Optical density at 600 nm (OD_600_) was monitored at regular intervals (every 4 or 2 h depending on the growth rate). Sampling was performed in the lag phase, mid-exponential phase and early stationary phase, according to OD_600_ based growth curves. For DOC quantification and DOM characterization 10 and 20 ml, respectively, were passed through a pre-rinsed polyether sulfone (PES) 0.2 μm pore size filter (Minisart, Sartorius, Göttingen, Germany), acidified to pH 2 with HCl (25%, p.a., Carl Roth, Germany) and stored at 4°C in the dark until further treatment. For substrate quantification, 5 ml were filtered (PES 0.2 μm pore size, Minisart, Sartorius, Göttingen, Germany) and stored at -20°C until analysis.

### Quantification of Substrate Concentration

Succinate and acetate concentrations in the treatments were determined by high pressure liquid chromatography (HPLC; Sykam, Fürstenfeldbruck, Germany) equipped with an Aminex HPX-87H column (Biorad, München, Germany) according to Graue et al. (2012). Detection limit was 1.5 μM acetate/succinate.

Glutamic acid concentrations were determined using HPLC after precolumn derivatization with orthophtaldialdehyde (Lunau et al., 2006) and concentrations of glucose by HPLC and pulsed amperometric detection after desalting (Hahnke et al., 2013) with a detection limit of 0.5 nM glutamic acid and 1.5 nM glucose, respectively.

### Bacterial Growth and Substrate Consumption

The growth of *D. shibae* and *P. inhibens* was assessed as optical density at 600 nm. The two bacteria had similar growth yield (approximately 1.0 OD_600_) except for *P. inhibens* growing on glutamate, which reached almost 2.0 OD_600_. Sampling points were decided for based on optical density, but substrate utilization was also quantified. The leftover portion of substrate at a given sampling point is reported in Supplementary Table S1.

In some cases, the sampling of the exponential phase took place before or after half of the substrate was consumed. However, the respective samples were all taken while the optical density was increasing exponentially, and therefore we consider these samples as taken during exponential phase. Given that in most set-ups, all substrate was used at the time of the stationary phase sampling, we assume that the limiting factor of our incubations was indeed the carbon source. For further details on incubation setup see Wienhausen et al. (2017).

### DOC Quantification and DOM Extraction

Dissolved organic carbon (DOC) was analyzed on a Shimadzu TOC-VCPH total organic carbon analyzer equipped with an autosampler ASI-V via high temperature catalytic combustion (Qian and Mopper, 1996). Accuracy of the method was tested using replicate measurements of Deep Atlantic Seawater Reference material (DSR, D.A. Hansell, University of Miami, Miami, FL, United States), which deviated on average < 5%. Standard deviation of biological triplicates was ±0.8 – 3%.

In order to desalt the samples and concentrate the DOM, we used solid phase extraction (Dittmar et al., 2008). Twenty milliliter of filtered and acidified sample ran through Varian Bond Elut PPL 100 mg cartridges (Agilent, United States) by gravity. After extraction, cartridges were rinsed with acidified ultrapure water (pH 2, HCl 25%, p.a., Carl Roth, Germany) to remove remaining salt. The resin was dried with Argon gas and eluted with 1 ml of methanol (HPLC-grade, Sigma-Aldrich, United States). The carbon concentration in the extracts was determined on the DOC analyzer as described before (Qian and Mopper, 1996), after extract aliquots were dried and dissolved in ultrapure water. Extraction efficiency with respect to carbon increased over the course of the experiment (from < 1% to 31%). The very low extraction efficiency at the beginning of the incubations mainly reflects the high carbon concentration due to the added substrate, which is mostly not extracted. Additionally the molecular composition of the exometabolites affects the extraction efficiencies, e.g., colloidal material and monomers are not well retained on the cartridges used (Hawkes et al., 2016). Procedural blanks were performed by running acidified ultra-pure water instead of sample. A mass balance with respect to DOC was done exemplarily for the incubations of *P. inhibens* growing on acetate (Supplementary Figure S1). At the beginning of the incubation most of the DOC was bound in the substrate that was added as carbon source. In exponential phase, still 96% of DOC was identified as acetate, 0.2% was SPE-DOC and 3% was out of our analytical window in terms of molecular characterization. For the final time point, acetate was below detection limit, 23.5% of the DOC was solid phase extracted, 9.8% was part of hydrolysable carbohydrates and 66% remained uncharacterized on the molecular level. Amino acids were below detection limit at all time points except stationary phase. The fraction of SPE-DOC increased over the course of the incubation, as well as the fraction of DOC that escaped molecular characterization, suggesting that the actual chemodiversity of the exometabolomes is even greater than the chemodiversity described in this study which is based on molecular characterization of the SPE-DOC fraction.

### DOM Characterization

Ultrahigh-resolution mass spectrometry via the Fourier transform ion cyclotron resonance (FT-ICR-MS) technique was performed on a Bruker Solarix 15 Tesla FT-ICR-MS (Bruker Daltonik GmbH, Bremen, Germany). Electrospray ionization was used in negative mode. Samples from different time points differed in DOM concentration, which could result in differential detection of individual masses. To avoid such artifacts, samples from lag, exponential and stationary phase were adjusted to 10 ppm DOC prior to analysis, in a carrier of ultrapure water and methanol (HPLC-grade, Sigma-Aldrich, United States) in equal parts. Five hundred scans were accumulated per run in a mass window of 92–2000 Da. The spectra were calibrated internally using Bruker Daltonics Data Analysis software package and processed using in-house MATLAB routines. The sum of all intensities detected with the FT-ICR-MS [total ion current (TIC), which is proportional to the amount of injected DOC] was calculated for each sample. TICs of all samples were in the same order of magnitude, which is a prerequisite for direct comparison. To separate analyte peaks from instrument noise we applied the method detection limit (MDL) described by Riedel and Dittmar (2014). Additionally, known contaminants were removed from the dataset. All masses present in the negative controls (sterile incubations) were removed from the data set. Masses present at the initial time point were kept only if their relative intensity increased in any of the following time points. Molecular formulas (MF) were assigned following the procedure described by Koch et al. (2007) with maximum elemental abundances of C_n_H_n_O_n_N_4_S_1_. The numbers reported here for the richness of the exometabolomes (number of species, in this case molecular masses) are higher than those reported by Wienhausen et al. (2017), first of all because they report number of MF, whereas we report number of detected masses. Secondly, the minimum detection limit (MDL) threshold set was less astringent for this study, therefore more masses were used to study the trends. Wienhausen et al. (2017) applied a more targeted approach, including fragmentation with MS/MS of peaks with high relative intensity. The general trends in chemical composition and chemodiversity were the same for both data sets.

Masses detected in at least two out of three biological replicates were included in further data analysis. The threshold of two out of three occurrences was chosen because the intention was not to artificially make the replicates identical to each other, but rather to observe the natural variability among the replicates and to assess the chemodiversity revealed by replicate analyses. We tested for the effect of including masses occurring in two out of three replicates versus considering occurrence in all three replicates. Even though half of the masses did not meet the criteria, i.e., were present in 2 out of 3 but not in all 3, we observed the same general trends in chemical composition (Principal component analysis; data not shown).

### Principal Component Analysis (PCA)

Results from FT-ICR-MS analyses were summarized in a table containing the detected masses with their corresponding signal intensities for each sample. After removing known contaminants and noise and applying thresholds (peaks present in at least two biological replicates), the signal intensities were normalized to relative abundance by dividing all peak intensities of one sample by the total sum of intensities in the sample and multiplying by 100. Additionally, standardization by *Z*-scores was done prior to the PCA. The PCA was performed on the normalized and standardized data with the software R version 3.3.1 (R core Team 2016) using the function “prcomp”. The significance of compositional change according to time and substrate was corroborated by a permutational multivariate analysis of variance using distance matrices. Both factors were significant with a *p*-value of < 0.001 (^∗∗∗^) for both strains. The function used was adonis() from the R package “vegan” using as factors time and substrate and 999 permutations.

### Diversity Indices for Exometabolomes

The diversity indices were calculated based on the detected molecular masses using MATLAB (Version 2015b, The MathWorks, Inc., Natick, MA, United States). The Shannon index (Shannon, 1948) considers both the richness (number of masses) and the evenness (how these molecular masses are distributed in terms of relative abundance) (Pielou, 1967). Pielou’s evenness (Pielou, 1967) was derived by dividing the Shannon index by the natural logarithm of the number of masses. These diversity indices are traditionally applied to assess biodiversity. In the case of chemodiversity species are detected molecular masses, which, similar to OTUs (operation taxonomic unit) in microbial diversity which represent several members, might represent several isomers (Hertkorn et al., 2008; Zark et al., 2017).

Functional diversity quantifies the variety of biological functions carried out by a community. In the context of chemical mixtures we assume that compounds with similar chemical properties react in similar ways (Mentges et al., 2017). The index was calculated using Rao’s entropy (Rao, 1982). The chemical properties used to calculate the index can vary, for this study, functional diversity was derived based on the number of nitrogen atoms, H/C ratios and mass range of masses with MF assigned. For the entire set of masses detected, regardless of formulae assignment, functional diversity as a function of mass was calculated.

We derived the chemodiversity of combinations of all the exometabolomes characterized in this study. Due to the high number of possible combinations (262,143), it is impossible to mix these samples physically, i.e., test *in vitro.* Therefore, we performed this analysis *in silico*, i.e., we derived the cumulative chemodiversity of exometabolome samples computationally by averaging the signal intensities over combinations of 1, 2, 3… 18 samples (2 strains × 3 substrates × 3 growth stages). Two approaches for subsequent molecular mass selection were tested. In the first approach we included all molecular masses that showed signal intensity > 0 in any of the samples. This approach yields the maximum observable chemodiversity for the cumulative sum of exometabolomes (“presence/absence based”). To mimic the effects of physically mixing, measuring and processing a pooled sample, we used a second data processing approach. We averaged the signal intensity over each set of samples and additionally applied the original MDL for each peak (“corrected with MDL”). This modified processing of the data strongly reduced the number of molecular masses in the cumulative sum of exometabolomes, as non-common molecular masses were excluded due to low average intensities (for a conceptual figure of the two approaches see Supplementary Figure S2).

### Marine DOM Representative

North Equatorial Pacific Intermediate Water (NEqPIW) (Bostock et al., 2010) is one of the oldest water masses in the oceans. A sample from this water mass is used as an “in house” reference representative of old and recalcitrant marine DOM. It was retrieved at the Natural Energy Laboratory of Hawaii Authority in 2009 (Green et al., 2014) and solid phase extracted with the same method described above for our samples (Dittmar et al., 2008). Extraction efficiency was 61 ± 3% with respect to carbon (Green et al., 2014). The NEqPIW sample was analyzed on the FT-ICR-MS repeatedly under the same conditions as the exometabolomes. All data analysis steps described for the exometabolomes were performed in parallel for 18 measurements of this reference sample.

## Results

### Molecular Characterization of Bacterial Exometabolomes

**FIGURE 1:**
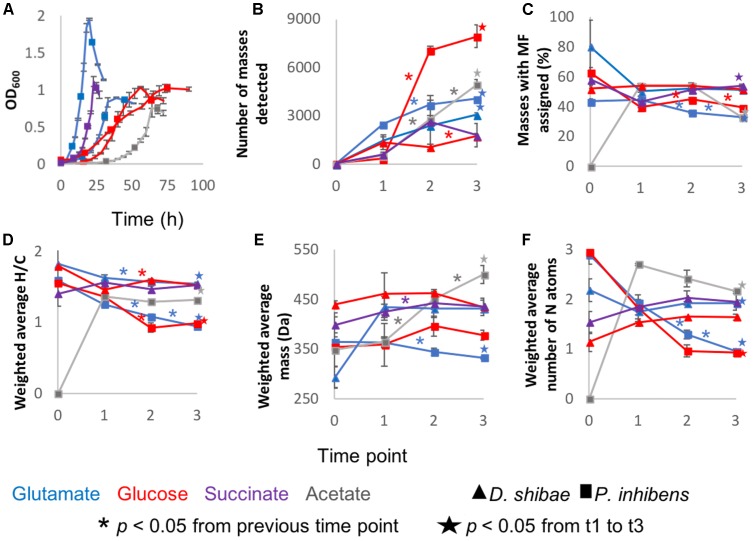
General description of the incubations: Averages of the three biological replicates of **(A)** Optical density measured at 600 nm wavelength. **(B)** Average number of detected masses in at least two biological replicates. **(C)** From the detected masses, the percentage to which molecular formulas (MF) were assigned. **(D)** Intensity-weighted average elemental ratios of hydrogen and carbon **(E)** Intensity-weighted average size of the masses detected via FT-ICR-MS. The reported number is the mean of the size averages of the three biological replicates **(F)** Intensity-weighted average of nitrogen atoms. Error bars represent standard deviation among the biological triplicates. The ^∗^ are added in the color coding of the substrate above the line to which they correspond and indicate that the difference between those two time points is statistically significant with *p* < 0.05. The star, also color coded, means that the difference between time points 1 and 3 is statistically significant with *p* < 0.05 and it is depicted above the final time point.

Pure cultures of *P. inhibens* and *D. shibae* were sampled at different growth stages determined by optical density (Figure 1A). Thousands of molecular masses were detected via FT-ICR-MS. Most incubations followed the same trend, starting at lag phase with a lower number of masses, i.e., richness, that increased rapidly. At the initial time point, richness was low but always higher than zero. These compounds were probably transferred with the original inoculum, but considered as they increased in relative abundance during the growth of the cultures. The number of masses increased continuously, and the maximum number was detected at the last sampling point at stationary phase (Figure 1B), with the exception of *D. shibae* growing on glucose and succinate.

The intensity-weighted average size of the masses detected per substrate and sampling point during the incubation was 292 – 499 Da. The average mass remained relatively constant from lag phase to stationary phase in *D. shibae* incubations. The incubations of *P. inhibens* showed different trends; growing on acetate the average size increased over time, whereas on glucose and glutamate it decreased after exponential and lag phase, respectively (Figure 1E).

Molecular formulae were assigned to 33 – 54% of the molecular masses detected via FT-ICR-MS. For the initial time points, this proportion was higher, due to the low number of molecular masses detected (Figure 1C). The intensity-weighted average nitrogen atoms per MF stayed relatively constant over time in *D. shibae* incubations, whereas for *P. inhibens* the number decreased over time (Figure 1F), while H/C ratios decreased over time in all incubations (Figure 1D).

**FIGURE 2:**
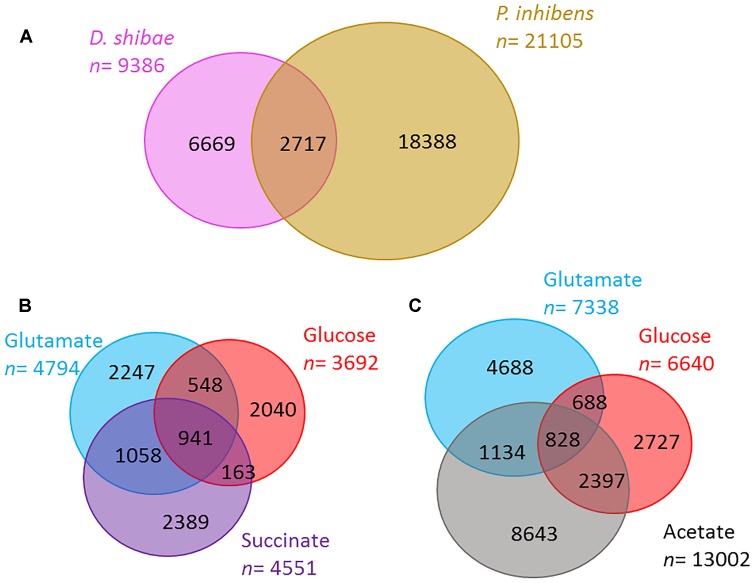
Richness of exometabolomes. **(A)** The number of molecular masses detected in the exometabolome of *D. shibae* (pink) and *P. inhibens* (brown) via FT-ICR-MS. **(B,C)** Number of molecular masses detected in the exometabolome of *D. shibae* **(B)** and *P. inhibens* **(C)** growing on different substrates (glutamate in blue, glucose in red, succinate in purple, and acetate in gray). Numbers reflect molecular masses detected in at least two biological replicates.

In the total exometabolome of each species that includes all substrates and time points tested, we detected 9,386 molecular masses for *D. shibae* and 21,105 in *P. inhibens* (molecular masses detected at any time point in at least two out of three replicates). Of these detected masses, 71 and 87% were unique to one of the strains, *D. shibae* and *P. inhibens*, respectively; the rest was shared between the two (Figure 2A). In *D. shibae* (Figure 2B) 10% of the masses were shared between the three substrates, for *P. inhibens* 4% (Figure 2C). The total ion current (TIC) or sum of intensities of the raw spectra was comparable among all samples. Based on the processed data after removal of noise, contamination and masses present in less than two biological replicates, the TIC of *P. inhibens* was higher than that of *D. shibae*. We excluded that the higher richness in the exometabolome of *P. inhibens* was a result of the higher TIC in the processed data by successively adding the intensities of masses found in the *P. inhibens* exometabolome, starting with the most intense peaks, until the sum was equal to the TIC of *D. shibae* exometabolomes and in all cases, the number of masses was still higher than those in *D. shibae*.

**FIGURE 3:**
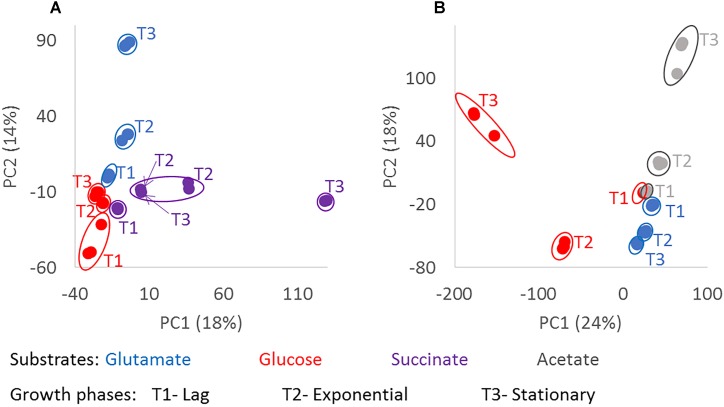
Chemodiversity of exometabolomes diverging with growth phase and substrate. Principle component analysis was done from the normalized intensities of the detected molecular masses in the exometabolome of *D. shibae* **(A)** and *P. inhibens* **(B)**. Masses detected in at least two biological replicates were considered. The color code indicates the substrate used as carbon source by the bacteria; glutamate in blue, glucose in red, succinate in purple and acetate in gray; where T1 is lag phase, T2 is exponential phase, and T3 is stationary phase. The composition of the exometabolomes of both strains changed significantly with *p*-value < 0.001 (^∗∗∗^) for growth phase and substrate.

Principal component analysis using the masses detected by FT-ICR-MS and their normalized relative intensities revealed differences in the chemical composition of the exometabolomes, based on the substrate the bacteria were utilizing and the growth phase of the bacteria at the given sampling point (Figure 3). In general, biological replicates grouped together in the PCA space comprised within the first two components. Only one replicate of the stationary phase of *D. shibae* growing on succinate located closer to the exponential phase (Figure 3A). The biological replicate from which this exometabolome was analyzed had a higher optical density at exponential growth phase than the other two replicates, which may explain the differences in composition. The exometabolome of *P. inhibens* growing on glutamate (Figure 3B) did not exhibit great variability in the first two principal components, but separation among the time points was still visible and the replicates were almost identical. The third and fourth principal components accounted for 10 and 9% of the variability in *D. shibae* and 14 and 9% in *P. inhibens.* Using either combination among the first four principal components, the biological replicates always grouped together (data not shown), indicating that exometabolome composition and its characterization via FT-ICR-MS are highly reproducible.

### Assessing Chemodiversity

**FIGURE 4:**
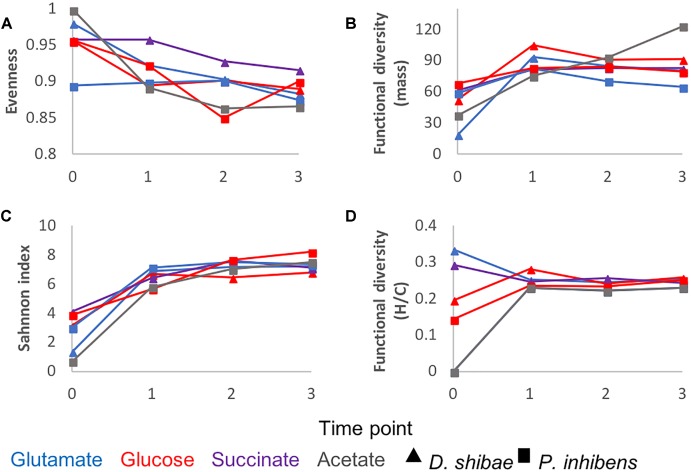
DOM chemodiversity described by different diversity measures: **(A)** evenness, **(B)** functional diversity of molecular mass **(C)** Shannon diversity index, **(D)** functional diversity of H/C ratios. Triangles refer to incubations of *D. shibae* and squares to incubations of *P. inhibens*. The colors refer to the substrate used by the bacteria as carbon source: Blue for glutamate, red for glucose, purple for succinate and gray for acetate. Diversity measures were calculated for each time point and substrate based on the average relative intensities of three biological replicates.

To further characterize the molecular composition of bacterial exometabolomes in terms of chemodiversity, ecological concepts and measures were applied to our dataset. Richness (Figure 1B) is the simplest of diversity measures, here represented by the number of detected masses. Evenness is a measure of the relative abundance distribution of species or molecular masses. It was higher at the beginning of the incubations, except for *P. inhibens* growing on glutamate, for which at the initial time point the evenness was lower (Figure 4A). The low evenness of this sample was driven by a few masses with high relative abundance. The Shannon index combines information on richness and evenness. It followed the same trends as richness in all incubations (Figure 4C).

The functional diversity calculated as a function of molecular mass increased over time in the exometabolome of *P. inhibens* growing on acetate (Figure 4B). In all other incubations functional diversity (mass) increased from the starting point to the lag phase and remained relatively constant until the end of the incubations (Figure 4B).

### Chemodiversity of Bacterial Exometabolomes and Marine DOM

**FIGURE 5:**
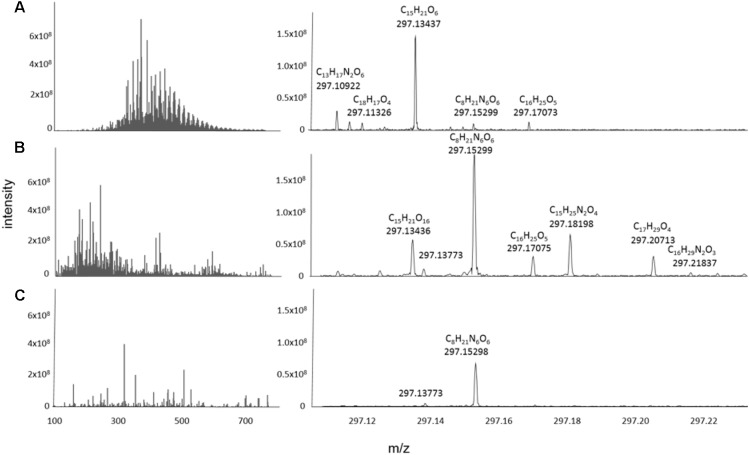
Representative mass spectra reconstructed from the processed data of **(A)** North Equatorial Pacific Intermediate Water (NEqPIW), **(B)** *D. shibae* in stationary phase growing on glucose **(C)** *P. inhibens* in stationary phase growing on glucose. Total ion current (the sum of intensities of the processed data) is 9.5 × 10^10^ for NEqPIW, 1.65 × 10^10^ for *D. shibae* and 1.07 × 10^11^ for *P. inhibens*. The latter is distributed among more peaks and thus the individual peaks have lower relative intensity than those of *D. shibae.*

The FT-ICR mass spectra of the exometabolomes were substantially different from those of marine DOM samples (Figure 5). Typically, the mass-intensity distribution of marine DOM is bell shaped and the highest relative intensities occur at around 425 m/z (e.g., Osterholz et al., 2015). Exometabolome spectra showed a different abundance distribution and the peaks appeared to be randomly distributed over a mass range of 100–550 Da. The proportion of peaks with assigned MF was 73% for marine DOM samples, and 42% for the exometabolome samples.

**FIGURE 6:**
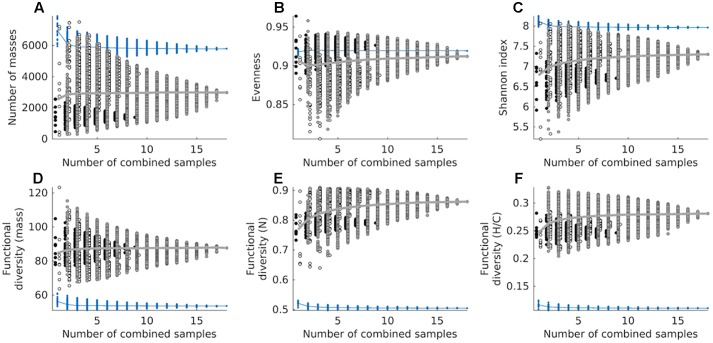
Cumulative chemodiversity of exometabolomes and marine DOM samples corrected by MDL, assessed as: **(A)** the number of molecular masses (richness), **(B)** Pielou’s evenness, **(C)** the Shannon index and **(D)** functional diversity of molecular mass, **(E)** functional diversity of number of N atoms, and **(F)** functional diversity of H/C ratios. Diversity measures **(A–D)** are based on detected masses, whereas **(E,F)** are based on assigned MF. Note that we added a small set-off between samples from different strains to minimize overlapping. A total of 262,143 combinations were tested for both datasets (exometabolomes and marine DOM). Circles filled black represent combinations of *D. shibae* exometabolomes exclusively, circles filled white represent combinations of *P. inhibens* exometabolomes exclusively, and circles filled gray represent combinations of exometabolomes from both strains. The mean of exometabolomes is given as a gray line. Blue dots represent combinations from repeated measurements of the North Equatorial Pacific Intermediate Water sample, its mean is given as a blue line.

We estimated the chemodiversity of the combined exometabolomes from the cumulative sum of exometabolomes in an *in silico* experiment. The cumulative sum of 18 exometabolomes had 27,774 masses (presence/absence based) (Supplementary Figure S3A). However when we mimicked the physical mixing and measuring of samples (simulated *in silico* by correcting with MDL) the richness of the sum of 18 exometabolomes was lower than that of marine DOM, with a total of 2982 masses detected (Figure 6A). Some combinations of 2, 3, 4, and 5 exometabolomes showed higher richness than marine DOM. The average number of masses detected in the marine DOM sample was 6970. This number dropped to 5793 when a cumulative sum of 18 deep water measurements was considered (corrected with MDL), as for the combined exometabolomes. On average, the evenness of combined exometabolomes was lower than that of marine DOM. However, the more exometabolomes were combined, the closer this value approached marine DOM evenness (Figure 6B). The functional diversity of the exometabolomes was higher than that of marine DOM, regardless of the data processing approach (Figure 6D–F). Note that all panels from Figure 6 are product of the MDL-corrected approach. Results from the presence/absence approach are displayed in Supplementary Figure S4.

## Discussion

### Exometabolome Chemodiversity of Single Strains

The chemodiversity of the exometabolomes of single strains was very high. This was expected considering previous studies, where thousands of molecular masses were detected in bacterial incubations using ultra-high resolution mass spectrometry (Rosselló-Mora et al., 2008; Romano et al., 2014; Fiore et al., 2015). We systematically confirmed that the composition of the exometabolome is affected by the physiological state of the strain, including growth phase and substrate used for assimilation.

The reason why the exometabolome of bacterial species is so diverse has not yet been elucidated. During the unfolding of metabolic processes errors can occur, resulting in “paralogous metabolism.” This could be a way to dispose potentially toxic variants of a metabolite and/or to find novel pathways that could evolve into a beneficial function (Danchin and Sekowska, 2014). This, together with overflow metabolism due to abundance of carbon source (Paczia et al., 2012; Romano et al., 2014), potentially increases the chemodiversity of metabolomes and could be considered a partial source of the chemodiversity we found. However, the high reproducibility in our experiments and the fact that independent biological replicates follow the same tendencies regarding composition of exometabolomes suggests that these compounds are not randomly excreted but rather are linked to prevailing metabolism of cells, as well as to gene expression, however this needs to be further analyzed.

Genome size and degree of streamlining, often correlates with the lifestyle of a bacterial group, as well as with their metabolic capabilities (Giovannoni et al., 2014). Despite the fact that their genomes have similar sizes (4.4 and 4.2 Mbp) the sizes of the exometabolomes of *D. shibae* and *P. inhibens* differed more than twofold (9,386 and 21,105 detected molecular masses, respectively). The number of molecular masses detected in *D. shibae* incubations was lower than that in *P. inhibens*, yet the number of protein encoding genes is higher in *D. shibae* compared to *P. inhibens* (4194 and 3875) (KEGG, Kanehisa and Goto, 2000). From our data, there is no indication that the genome size or the number of protein encoding genes directly correlates with the richness of the exometabolome. Possibly, the very elaborate secondary metabolism of *P. inhibens* leads to a larger exometabolome (Thole et al., 2012; Wilson et al., 2016).

The growth of auxotrophic organisms in the ocean relies highly on the supply of metabolites by co-occurring microbes, which have “leaky” metabolic pathways (Morris et al., 2012). This mutualistic relationship, between auxotrophs and helpers, drives processes of co-evolution among the players (Mas et al., 2016). The two bacterial species analyzed in this study have different lifestyles; *P. inhibens* was isolated from the Atlantic coast of North Western Spain (Ruiz-Ponte et al., 1998). It produces several secondary metabolites, including the antibiotic tropodithietic acid (TDA), roseobacticides, several acyl homoserine lactones (AHLs), B vitamins and it is known to attach to surfaces and form biofilms (Thole et al., 2012; Wilson et al., 2016; Wienhausen et al., 2017). Biofilm production requires the excretion of a range of specific organic compounds, particularly polysaccharides. This lifestyle could account for the fact that more masses were detected in its exometabolome. *D. shibae*, isolated from the surface of a dinoflagellate, is capable of performing anoxygenic photosynthesis, as well as synthesizing a series of B vitamins and AHLs, but no antibiotics (Wagner-Döbler et al., 2010; Wienhausen et al., 2017). These distinct capabilities are expected to strongly influence the exometabolome, as most of these metabolites work outside of the cell and are aimed for interaction with other cells of the same or other species. Both, *P. inhibens* and *D. shibae* are likely to exchange metabolites among members of the same species as well as with other organisms in their natural environment, contributing to the marketplace of metabolites in the marine ecosystems (Wienhausen et al., 2017).

Most of the MF detected are not predicted by the genome and/or could not be matched to any metabolite database, consistent with previous studies (Romano et al., 2014; Wienhausen et al., 2017). Metabolites, especially when they have not been predicted by an organism’s genome, are likely to be excreted and have ecological relevance (Fiore et al., 2015). These metabolites could be the result of errors in the unfolding of core metabolites, thus being part of the paralogous metabolism (Danchin and Sekowska, 2014) or intermediates of different metabolic pathways that are released because of metabolic dead ends or due to physiological conditions like excess of carbon (i.e., “over-flow metabolism”) (Paczia et al., 2012). Additionally, there might be a bias in the databases favoring the metabolic annotation of pathogenic bacteria rather than environmentally relevant bacteria. Databases occasionally contain information of genes wrongly annotated or lack compounds from biosynthetic pathways, decreasing the number of identified molecules in metabolomics studies (Romano et al., 2014). The detected masses that could indeed be related to the genome indicate that these two marine organisms may play important roles supplying growth factors and biosynthetic precursors to the environment, which could be used by other marine organisms (Wienhausen et al., 2017). Even if some of the masses detected via FT-ICR-MS are metabolic waste, without any particular function for the bacteria that produced it, it is remarkable that a single bacterial species has the potential to release thousands of molecular masses outside the cell and possibly into the ocean as has been indicated by the match of a fraction of MF released by the two model strains to MF produced during natural and naturally-derived phytoplankton blooms (Wienhausen et al., 2017).

### Linking the Chemodiversity of Bacterial Exometabolomes to Marine DOM

Extremely high chemodiversity and consequently very low substrate concentration, is discussed as one of the reasons for the long-term stability of DOM in the oceans (Barber, 1968; Arrieta et al., 2015; Dittmar, 2015). Since the source of DOM is presumably microbial (Dittmar and Stubbins, 2014) and marine bacteria play an important role in the transformation of phytoplankton-derived organic matter (Jiao et al., 2010; Paul et al., 2013; Lechtenfeld et al., 2015; Osterholz et al., 2015), we hypothesize that metabolic functions controlling the chemodiversity of marine bacterial exometabolomes are also instrumental in shaping the chemodiversity of the ocean’s geometabolome, i.e., marine DOM. We found that the cumulative sum of exometabolomes is highly diverse and in some cases even more diverse than marine DOM. About 15% of the 13,509 molecular formulae detected in exometabolomes and refractory oceanic DOM were shared, i.e., occurred in *Roseobacter* exometabolomes and in deep water samples. This overlap, although small supports our hypothesis that marine bacteria from the *Roseobacter* group are at least partly responsible for sustaining marine DOM chemodiversity and stability. There are also vast differences between the bacterial exometabolomes and the ocean’s geometabolome. Hence, further processes, presumably mostly microbial and consistent with the concept of the microbial carbon pump (Jiao et al., 2010), have to be involved to yield the typical pattern and structure of the marine geometabolome.

The presence/absence based cumulative sum of the exometabolomes shows that the richness in combined exometabolomes is enormous and potentially higher than that in marine DOM. However, due to characteristics of the method (e.g., semi-quantitative and subjective distinction of noise from signal), the interpretation of this data has to be done cautiously. The number of masses changes dramatically whether we correct the intensities of the computationally summed up samples by MDL or not. In the MDL corrected dataset, the cumulative average intensity of a molecular mass needs to exceed the MDL, otherwise it is excluded. Using this approach, the 18 exometabolomes have only 5 molecular masses in common, because a large proportion of masses was detected at low average signal intensities and is consequently excluded during data processing. In contrast, the 18 measurements of marine DOM share 3917 masses and consequently a higher fraction of these molecular masses remains after data processing. Thus, by summing up the exometabolomes *in silico* without correcting with MDL, we detect the “rare chemosphere” that otherwise would stay hidden when we measure a sample made by physically pooling several samples or measuring environmental samples.

This strong dependency of the number of masses on data processing indicates that richness is not a robust indicator of chemodiversity for intercomparison between studies, because it can be biased by the sensitivity and detection limit of the chemical analysis. As the Shannon index is mainly driven by richness in this set of samples, it is thus also not robust. In contrast, functional diversity indices have been shown to be largely robust to richness effects, making them suitable to compare DOM samples of different origin (Mentges et al., 2017). The functional diversity in the exometabolomes is higher than in marine DOM regardless of the data processing approach. Thus, compared to marine DOM, in the exometabolome a larger fraction of signal intensities is made up by compounds with relatively high or low masses, H/C ratios and number of N atoms. It is important to mention that this holds despite the fact that the mass range of all compounds identified in the exometabolome versus marine DOM compound is similar; the same applies for the range of H/C ratios and the range of number of N atoms.

In natural environments, diverse microbial communities are interwoven with diverse DOM in complex interactive networks. Osterholz et al. (2015) studied the diversification of microbial DOM in incubations, where phytoplankton and bacterial communities from the North Sea interacted for 3 years. The chemodiversity of the resulting microbial DOM (combined microbial exometabolome) has been assessed with the functional diversity indices also applied in this study (Mentges et al., 2017). The functional diversity of H/C ratios of the microbial DOM is largely between 0.12 and 0.16 and decreasing over time of incubation (Mentges et al., 2017). The functional diversity of H/C ratios of the “younger” material approaches that of marine DOM over time. The functional diversity of H/C ratios of our combined bacterial exometabolomes (Figure 6F) is about double the functional diversity of the microbial DOM studied in Mentges et al. (2017). The same applies to the individual strains: the individual exometabolomes reach a higher functional diversity than observed in the incubations with complex microbial communities (Figure 4). The exometabolomes of individual bacterial strains are highly functionally diverse. With increasing trophic interactions and biotic and abiotic transformations the functional diversity of microbial DOM approaches that of marine DOM. The number of MF in the more complex incubations increased over time of incubation from about a 1000–6000 (Mentges et al., 2017). Similar to the functional diversity, the richness is more representative for bacterial exometabolomes at the start of microbial incubations, but gets more similar to marine DOM with time. Our study provides further evidence for the potential role of microorganisms in the diversification of marine DOM. Given the vast genetic repertoire of natural microbial communities, the multitude of available substrates and the complex interactions in marine environments, we conclude that active microbial communities play a central role in producing and sustaining the observed chemodiversity of DOM in the ocean.

## Data Availability Statement

The dataset generated for this study can be found filtered for noise and normalized in PANGAEA (https://www.pangaea.de/) doi: https://doi.pangaea.de/10.1594/PANGAEA.892793.

## Author Contributions

BN-O designed and performed the experiments, analyzed and interpreted the data, and drafted and revised the intellectual content. GW designed and performed the experiments and revised the intellectual content. AM performed the *in silico* experiments and functional diversity calculations, and drafted and revised the intellectual content. JN, MS, and TD designed the experiments and revised the intellectual content.

## Conflict of Interest Statement

The authors declare that the research was conducted in the absence of any commercial or financial relationships that could be construed as a potential conflict of interest.

## References

[B1] Aiken G. R. Malcolm R. L. (1987). Molecular weight of aquatic fulvic acids by vapor pressure osmometry. *Geochim. Cosmochim. Acta* 51 2177–2184. 10.1016/0016-7037(87)90267-5

[B2] Allen J. Davey H. M. Broadhurst D. Heald J. K. Rowland J. J. Oliver S. G. (2003). High-throughput classification of yeast mutants for functional genomics using metabolic footprinting. *Nat. Biotechnol.* 21 692–696. 10.1038/nbt823 12740584

[B3] Arrieta J. M. Mayol E. Hansman R. L. Herndl G. J. Dittmar T. Duarte C. M. (2015). Dilution limits dissolved organic carbon utilization in the deep ocean. *Science* 348 331–333. 10.1126/science.1258955 25883355

[B4] Azam F. Malfatti F. (2007). Microbial structuring of marine ecosystems. *Nat. Rev. Microbiol.* 5 782–791. 10.1038/nrmicro1747 17853906

[B5] Barber R. T. (1968). Dissolved organic carbon from deep waters resists microbial oxidation. *Nature* 220 274–275. 10.1038/220274a0 5684857

[B6] Bauer J. E. Williams P. M. Druffel E. R. M. (1992). 14C activity of dissolved organic carbon fractions in the north-central Pacific and Sargasso Sea. *Nature* 357:667 10.1038/357667a0

[B7] Biebl H. Allgaier M. Tindall B. J. Koblizek M. Lünsdorf H. Pukall R. (2005). *Dinoroseobacter shibae* gen. nov., sp. nov., a new aerobic phototrophic bacterium isolated from dinoflagellates. *Int. J. Syst. Evol. Microbiol.* 55 1089–1096. 10.1016/B978-0-12-405940-5.00007-8 15879238

[B8] Bostock H. C. Opdyke B. N. Williams M. J. M. (2010). Characterising the intermediate depth waters of the Pacific Ocean using δ13C and other geochemical tracers. *Deep Sea Res. I Oceanogr. Res. Pap.* 57 847–859. 10.1099/ijs.0.63511-0 15879238

[B9] Buchan A. LeCleir G. R. Gulvik C. A. Gonzalez J. M. (2014). Master recyclers: features and functions of bacteria associated with phytoplankton blooms. *Nat. Rev. Microbiol.* 12 686–698. 10.1038/nrmicro3326 25134618

[B10] Carlson C. A. del Giorgio P. A. Herndl G. J. (2007). Microbes and the dissipation of energy and respiration: from cells to ecosystems. *Oceanography* 20 89–100. 10.1038/nrmicro3326 25134618

[B11] Danchin A. Sekowska A. (2014). The logic of metabolism and its fuzzy consequences. *Environ. Microbiol.* 16 19–28. 10.5670/oceanog.2007.52 24387040

[B12] Dittmar T. (2015). “Reasons behind the long-term stability of dissolved organic matter,” in *Biogeochemistry of Marine Dissolved Organic Matter*, eds Hansell D. A. Carlson C. A. (New York, NY: Elsevier Inc.), 369–388. 10.1111/1462-2920.12270

[B13] Dittmar T. Koch B. Hertkorn N. Kattner G. (2008). A simple and efficient method for the solid-phase extraction of dissolved organic matter (SPE-DOM) from seawater. *Limnol. Oceanogr. Methods* 6 230–235. 10.4319/lom.2008.6.230

[B14] Dittmar T. Stubbins A. (2014). “Dissolved organic matter in aquatic systems,” in *Treatise on Geochemistry (Second Edition)*, eds Falkowski P. G. Freeman K. H. (Oxford: Elsevier), 125–156. 10.1016/B978-0-08-095975-7.01010-X

[B15] Fiore C. L. Longnecker K. Kido Soule M. C. Kujawinski E. B. (2015). Release of ecologically relevant metabolites by the cyanobacterium *Synechococcus elongatus* CCMP 1631. *Environ. Microbiol.* 17 3949–3963. 10.1111/1462-2920.12899 25970745

[B16] Giovannoni S. J. Cameron Thrash J. Temperton B. (2014). Implications of streamlining theory for microbial ecology. *ISME J.* 8 1553–1565. 10.1038/ismej.2014.60 24739623 PMC4817614

[B17] Graue J. Engelen B. Cypionka H. (2012). Degradation of cyanobacterial biomass in anoxic tidal-flat sediments: a microcosm study of metabolic processes and community changes. *ISME J.* 6 660–669. 10.1038/ismej.2011.120 21918576 PMC3280128

[B18] Green N. W. Perdue E. M. Aiken G. R. Butler K. D. Chen H. Dittmar T. (2014). An intercomparison of three methods for the large-scale isolation of oceanic dissolved organic matter. *Mar. Chem.* 161 14–19. 10.1016/j.marchem.2014.01.012

[B19] Hahnke S. Sperling M. Langer T. Wichels A. Gerdts G. Beardsley C. (2013). Distinct seasonal growth patterns of the bacterium *Planktotalea frisia* in the North Sea and specific interaction with phytoplankton algae. *FEMS Microbiol. Ecol.* 86 185–199. 10.1111/1574-6941.12151 23711338

[B20] Hansell D. A. (2013). Recalcitrant dissolved organic carbon fractions. *Annu. Rev. Mar. Sci.* 5 421–445. 10.1146/annurev-marine-120710-100757 22881353

[B21] Hawkes J. A. Dittmar T. Patriarca C. Tranvik L. Bergquist J. (2016). Evaluation of the orbitrap mass spectrometer for the molecular fingerprinting analysis of natural dissolved organic matter. *Anal. Chem.* 88 7698–7704. 10.1021/acs.analchem.6b01624 27400998

[B22] Hedges J. I. Eglinton G. Hatcher P. G. Kirchman D. L. Arnosti C. Derenne S. (2000). The molecularly-uncharacterized component of nonliving organic matter in natural environments. *Organ. Geochem.* 31 945–958. 10.1016/S0146-6380(00)00096-6

[B23] Hertkorn N. Frommberger M. Witt M. Koch B. P. Schmitt-Kopplin P. Perdue E. M. (2008). Natural organic matter and the event horizon of mass spectrometry. *Anal. Chem.* 80 8908–8919. 10.1021/ac800464g 19551926

[B24] Jiao N. Herndl G. J. Hansell D. A. Benner R. Kattner G. Wilhelm S. W. (2010). Microbial production of recalcitrant dissolved organic matter: long-term carbon storage in the global ocean. *Nat. Rev. Microbiol.* 8 593–599. 10.1038/nrmicro2386 20601964

[B25] Kanehisa M. Goto S. (2000). KEGG: kyoto encyclopedia of genes and genomes. *Nucleic Acids Res.* 28 27–30. 10.1093/nar/28.1.27 10592173 PMC102409

[B26] Koch B. P. Dittmar T. Witt M. Kattner G. (2007). Fundamentals of molecular formula assignment to ultrahigh resolution mass data of natural organic matter. *Anal. Chem.* 79 1758–1763. 10.1021/ac061949s 17297983

[B27] Koch B. P. Kattner G. Witt M. Passow U. (2014). Molecular insights into the microbial formation of marine dissolved organic matter: recalcitrant or labile? *Biogeosciences* 11 4173–4190. 10.5194/bg-11-4173-2014

[B28] Kujawinski E. B. (2011). The impact of microbial metabolism on marine dissolved organic matter. *Annu. Rev. Mar. Sci.* 3 567–599. 10.1146/annurev-marine-120308-081003 21329217

[B29] Kujawinski E. B. Longnecker K. Blough N. V. Vecchio R. D. Finlay L. Kitner J. B. (2009). Identification of possible source markers in marine dissolved organic matter using ultrahigh resolution mass spectrometry. *Geochim. Cosmochim. Acta* 73 4384–4399. 10.1016/j.gca.2009.04.033

[B30] Landa M. Cottrell M. T. Kirchman D. L. Kaiser K. Medeiros P. M. Tremblay L. (2014). Phylogenetic and structural response of heterotrophic bacteria to dissolved organic matter of different chemical composition in a continuous culture study. *Environ. Microbiol.* 16 1668–1681. 10.1111/1462-2920.12242 24020678

[B31] Lechtenfeld O. J. Hertkorn N. Shen Y. Witt M. Benner R. (2015). Marine sequestration of carbon in bacterial metabolites. *Nat. Commun.* 6:6711. 10.1038/ncomms7711 25826720

[B32] Lunau M. Lemke A. Dellwig O. Simon M. (2006). Physical and biogeochemical controls of microaggregate dynamics in a tidally affected coastal ecosystem. *Limnol. Oceanogr.* 51 847–859. 10.4319/lo.2006.51.2.0847

[B33] Mapelli V. Olsson L. Nielsen J. (2008). Metabolic footprinting in microbiology: methods and applications in functional genomics and biotechnology. *Trends Biotechnol.* 26 490–497. 10.1016/j.tibtech.2008.05.008 18675480

[B34] Martens T. Heidorn T. Pukall R. Simon M. Tindall B. J. Brinkhoff T. (2006). Reclassification of *Roseobacter gallaeciensis* Ruiz-Ponte et al., 1998 as *Phaeobacter gallaeciensis* gen. nov., comb. nov., description of *Phaeobacter inhibens* sp. nov., reclassification of *Ruegeria algicola* (Lafay et al. 1995) Uchino et al. 1999 as *Marinovum algicola* gen. nov., comb. nov., and emended descriptions of the genera *Roseobacter, Ruegeria* and *Leisingera*. *Int. J. Syst. Evol. Microbiol.* 56 1293–1304. 10.1099/ijs.0.63724-0 16738106

[B35] Mas A. Jamshidi S. Lagadeuc Y. Eveillard D. Vandenkoornhuyse P. (2016). Beyond the black queen hypothesis. *ISME J.* 10 2085–2091. 10.1038/ismej.2016.22 26953598 PMC4989313

[B36] Mentges A. Feenders C. Seibt M. Blasius B. Dittmar T. (2017). Functional molecular diversity of marine dissolved organic matter is reduced during degradation. *Front. Mar. Sci.* 4:194 10.3389/fmars.2017.00194

[B37] Morris J. J. Lenski R. E. Zinser E. R. (2012). The black queen hypothesis: evolution of dependencies through adaptive gene loss. *mBio* 3:e0036-12. 10.1128/mBio.00036-12 22448042 PMC3315703

[B38] Ogawa H. Amagai Y. Koike I. Kaiser K. Benner R. (2001). Production of refractory dissolved organic matter by bacteria. *Science* 292 917–920. 10.1126/science.1057627 11340202

[B39] Oliver S. G. Winson M. K. Kell D. B. Baganz F. (1998). Systematic functional analysis of the yeast genome. *Trends Biotechnol.* 16 373–378. 10.1016/S0167-7799(98)01214-1 9744112

[B40] Osterholz H. Niggemann J. Giebel H. A. Simon M. Dittmar T. (2015). Inefficient microbial production of refractory dissolved organic matter in the ocean. *Nat. Commun.* 6:7422. 10.1038/ncomms8422 26084883

[B41] Paczia N. Nilgen A. Lehmann T. Gätgens J. Wiecher W. Noack S. (2012). Extensive exometabolome analysis reveals extended overflow metabolism in various microorganisms. *Microb. Cell Fact.* 11:122. 10.1186/1475-2859-11-122 22963408 PMC3526501

[B42] Paul C. Mausz M. A. Pohnert G. (2013). A co-culturing/metabolomics approach to investigate chemically mediated interactions of planktonic organisms reveals influence of bacteria on diatom metabolism. *Metabolomics* 9 349–359. 10.1007/s11306-012-0453-1

[B43] Pielou E. C. (1967). The measurement of diversity in different types of biological collections. *J. Theor. Biol.* 15:177 10.1016/0022-5193(67)90048-3

[B44] Qian J. Mopper K. (1996). Automated high-performance, high-temperature combustion total organic carbon analyzer. *Anal. Chem.* 68 3090–3097. 10.1021/ac960370z

[B45] Rao C. R. (1982). Diversity and dissimilarity coefficients: a unified approach. *Theor. Popul. Biol.* 21 24–43. 10.1016/0040-5809(82)90004-1

[B46] Riedel T. Dittmar T. (2014). A method detection limit for the analysis of natural organic matter via fourier transform ion cyclotron resonance mass spectrometry. *Anal. Chem.* 86 8376–8382. 10.1021/ac501946m 25068187

[B47] Romano S. Dittmar T. Bondarev V. Weber R. J. M. Viant M. R. Schulz-Vogt H. N. (2014). Exo-Metabolome of *Pseudovibrio* sp. FO-BEG1 Analyzed by ultra-high resolution mass spectrometry and the effect of phosphate limitation. *PLoS One* 9:e96038. 10.1371/journal.pone.0096038 24787987 PMC4008564

[B48] Rosselló-Mora R. Lucio M. Peña A. Brito-Echeverría J. López-López A. Valens-Vadell M. (2008). Metabolic evidence for biogeographic isolation of the extremophilic bacterium *Salinibacter ruber*. *ISME J.* 2:242. 10.1038/ismej.2007.93 18239610

[B49] Ruiz-Ponte C. Cilia V. Lambert C. Nicolas J. L. (1998). *Roseobacter gallaeciensis* sp. nov., a new marine bacterium isolated from rearings and collectors of the scallop *Pecten maximus. Int. J. Syst. Bacteriol.* 48(pt 2),537–542. 10.1099/00207713-48-2-537 9731295

[B50] Segev E. Wyche T. P. Kim K. H. Petersen J. Ellebrandt C. Vlamakis H. (2016). Dynamic metabolic exchange governs a marine algal-bacterial interaction. *eLife* 5:e17473. 10.7554/eLife.17473 27855786 PMC5148602

[B51] Shade A. (2017). Diversity is the question, not the answer. *ISME J.* 11 1–6. 10.1038/ismej.2016.118 27636395 PMC5421358

[B52] Shannon C. E. (1948). A mathematical theory of communication. *Bell. Syst. Technol. J.* 27 379–423. 10.1002/j.1538-7305.1948.tb01338.x

[B53] Simpson A. J. (2002). Determining the molecular weight, aggregation, structures and interactions of natural organic matter using diffusion ordered spectroscopy. *Magn. Reson. Chem.* 40 S72–S82. 10.1002/mrc.1106

[B54] Teeling H. Fuchs B. M. Becher D. Klockow C. Gardebrecht A. Bennke C. M. (2012). Substrate-controlled succession of marine bacterioplankton populations induced by a phytoplankton bloom. *Science* 336 608–611. 10.1126/science.1218344 22556258

[B55] Thole S. Kalhoefer D. Voget S. Berger M. Engelhardt T. Liesegang H. (2012). Phaeobacter gallaeciensis genomes from globally opposite locations reveal high similarity of adaptation to surface life. *ISME J.* 6 2229–2244. 10.1038/ismej.2012.62 22717884 PMC3504968

[B56] Tilman D. (2001). *Functional Diversity A2 – Levin, Simon Asher. Encyclopedia of Biodiversity*. New York, NY: Elsevier 109–120. 10.1016/B0-12-226865-2/00132-2

[B57] Wagner-Döbler I. Ballhausen B. Berger M. Brinkhoff T. Buchholz I. Bunk B. (2010). The complete genome sequence of the algal symbiont *Dinoroseobacter shibae*: a hitchhiker’s guide to life in the sea. *ISME J.* 4 61–77. 10.1038/ismej.2009.94 19741735

[B58] Wienhausen G. Noriega-Ortega B. E. Niggemann J. Dittmar T. Simon M. (2017). The exometabolome of two model strains of the roseobacter group: a marketplace of microbial metabolites. *Front. Microbiol.* 8:1985. 10.3389/fmicb.2017.01985 29075248 PMC5643483

[B59] Williams P. M. Oeschger H. Kinney P. (1969). Natural radiocarbon activity of the dissolved organic carbon in the north-east pacific ocean. *Nature* 224:256 10.1038/224256a0

[B60] Wilson M. Z. Wang R. Gitai Z. Seyedsayamdost M. R. (2016). Mode of action and resistance studies unveil new roles for tropodithietic acid as an anticancer agent and the γ-glutamyl cycle as a proton sink. *Proc. Natl. Acad. Sci. U.S.A.* 113 1630–1635. 10.1073/pnas.1518034113 26802120 PMC4760781

[B61] Zark M. Christoffers J. Dittmar T. (2017). Molecular properties of deep-sea dissolved organic matter are predictable by the central limit theorem: evidence from tandem FT-ICR-MS. *Mar. Chem.* 191(Suppl. C), 9–15. 10.1016/j.marchem.2017.02.005

[B62] Zech H. Thole S. Schreiber K. Kalhöfer D. Voget S. Brinkhoff T. (2009). Growth phase-dependent global protein and metabolite profiles of *Phaeobacter gallaeciensis* strain DSM 17395, a member of the marine Roseobacter-clade. *Proteomics* 9 3677–3697. 10.1002/pmic.200900120 19639587

