# Supplementary material for: Does the Chemodiversity of Bacterial Exometabolomes Sustain the Chemodiversity of Marine Dissolved Organic Matter?
Source: Front Microbiol. 2019 Feb 14;10:215. doi: 10.3389/fmicb.2019.00215 (PMC6382689; doi:10.3389/fmicb.2019.00215)
Supplement: Supplementary file 1 [file Data_Sheet_1.docx]

Supplementary Material

Does the chemodiversity of bacterial exometabolomes sustain the chemodiversity of marine dissolved organic matter?

**Beatriz E Noriega-Ortega*, Gerrit Wienhausen, Andrea Mentges, Thorsten Dittmar, Meinhard Simon and Jutta Niggemann**

*** Correspondence:**Beatriz E Noriega-Ortega
beatriz.elizabeth.noriega.ortega@uni-oldenburg.de

**
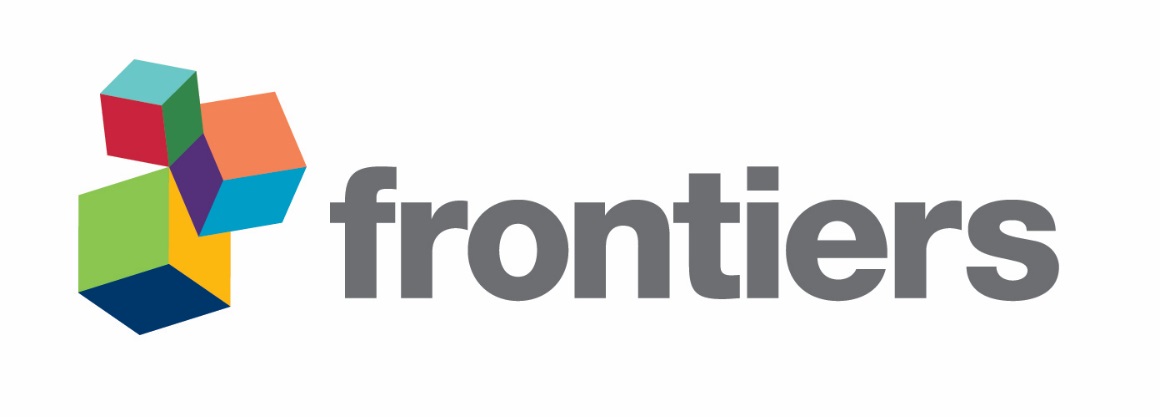
**

1,401 μmol 1,108 μmol 970 μmol 48 μmol

**Supplementary figure S1 |** Mass balance with respect to carbon of incubations of *P. inhibens* growing on acetate. All amino acids, carbohydrates and substrate quantified by HPLC, as well as solid phase extracted material were considered as a percentage of the total organic carbon measured in the incubations. The total amount of carbon given on top of the bars refers to the dissolved organic carbon quantified in a sample volume of 20 ml.


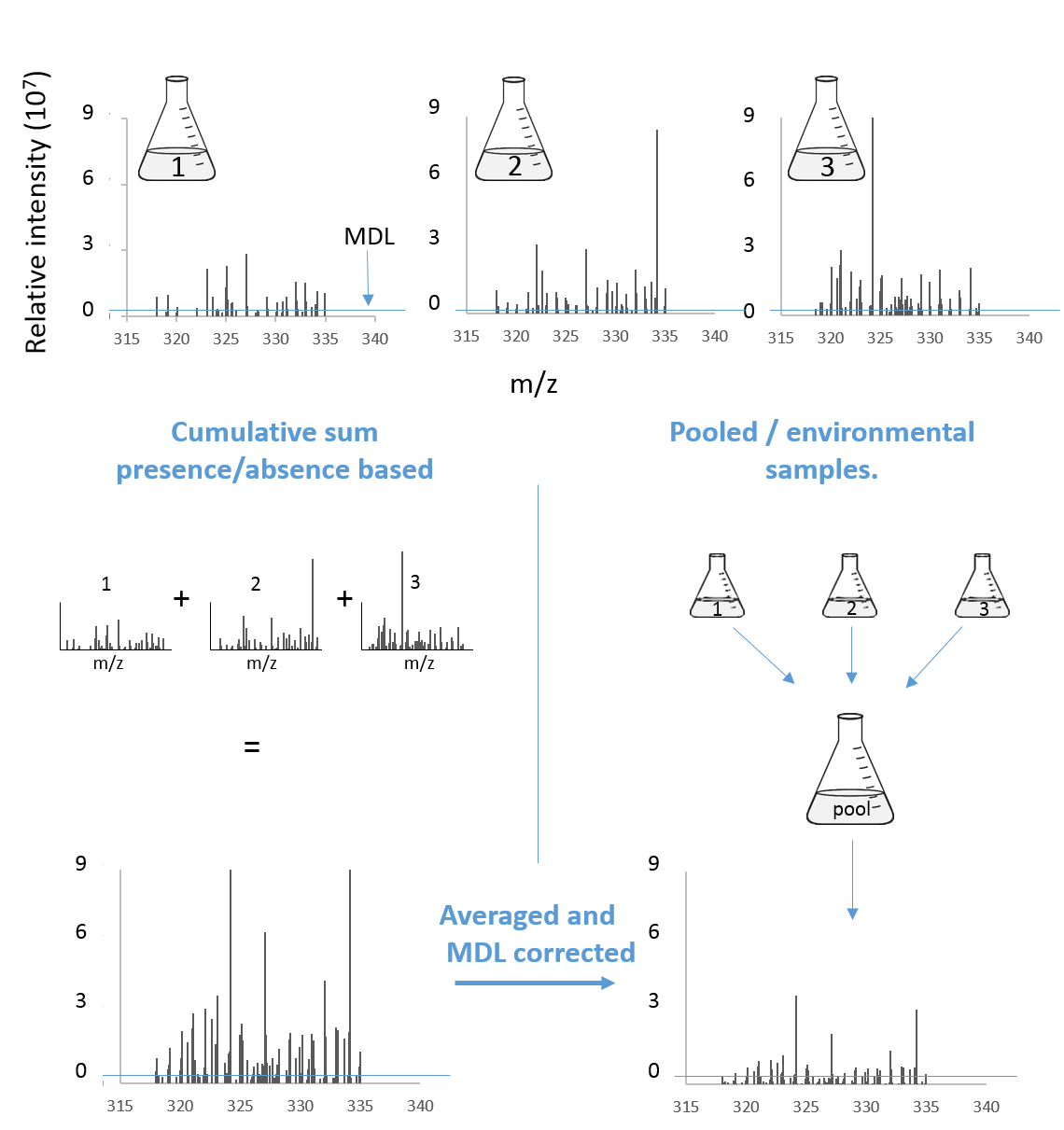


**Supplementary figure S2 |** The cumulative chemodiversity of exometabolome samples was derived by averaging the signal intensities over combinations of 1, 2, 3 ... 18 samples (2 strains x 3 substrates x 3 growth stages). Due to the high number of possible combinations (262,143), it is impossible to test *in vitro.* Two approaches for subsequent molecular mass selection were tested. In the first approach we included all molecular masses that showed signal intensity > 0 in any of the samples. This approach yields the maximum observable chemodiversity for the cumulative sum of exometabolomes (“presence/absence based”). To mimic the effects of physically mixing, measuring and processing a pooled sample, we used a different data processing approach. We averaged the signal intensity over each set of samples and additionally applied the original MDL for each peak (“corrected with MDL”). This modified processing of the data strongly reduced the number of molecular masses in the cumulative sum of exometabolomes, as non-common molecular masses were excluded due to averages that are lower than the MDL.


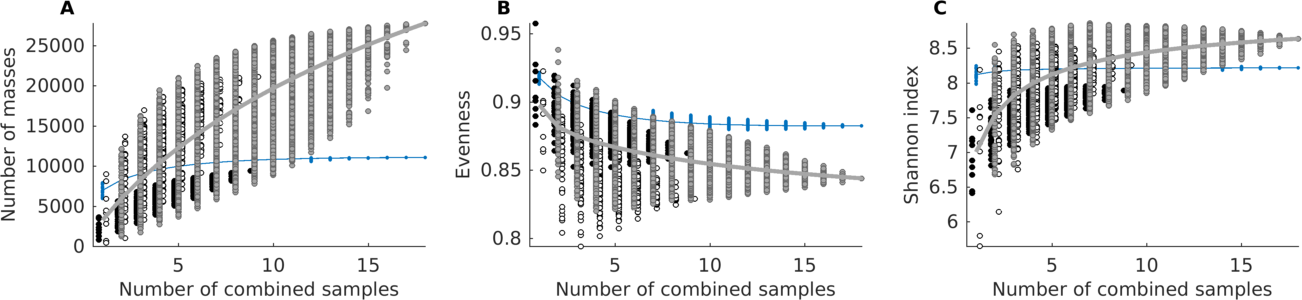


**Supplementary figure S3 |** Cumulative chemodiversity of exometabolomes and marine DOM samples. All intensities of the masses present in the samples were used to create the cumulative sum (presence/absence approach). Chemodiversity is quantified by **A)** the number of molecular masses (richness), **B)** Pielou's evenness, **C)** the Shannon index. Note that we added a small set-off between samples from different strains to minimize overlapping. A total of 262,143 combinations were tested for both datasets (exometabolomes and marine DOM). Circles filled black represent combinations made out of *D. shibae* exometabolomes exclusively. Circles filled white represent combinations made out of *P. inhibens* exometabolomes exclusively. Circles filled grey represent combinations of exometabolomes from both strains. The mean of exometabolomes is given as a grey line. Blue dots represent combinations from repeated measurements of the same deep-water sample, its mean is given as a blue line.


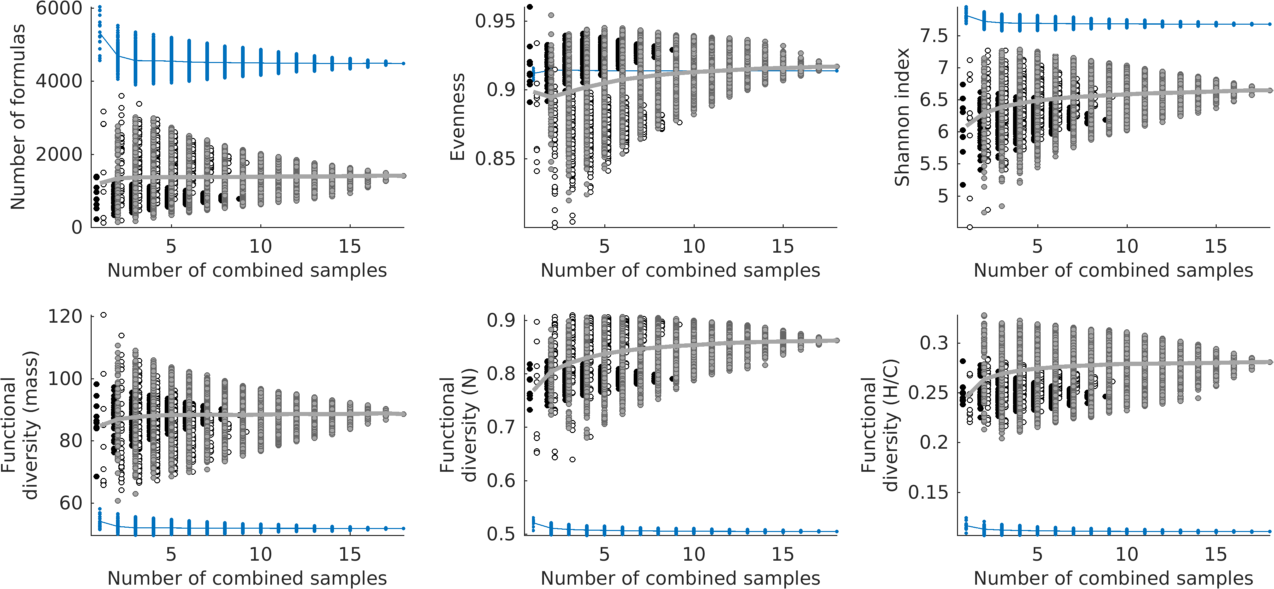


**Supplementary figure S4 |** Cumulative chemodiversity of exometabolomes and marine DOM samples using only masses with molecular formulae assignment. The cumulative sum was created by adding the intensities of the masses present, followed by the application of the method detection limit as a threshold (corrected with MDL). This step mimics *in silico* the physical pooling of samples prior to measurement in the mass spectrometer. Chemodiversity is quantified by **A)** the number of molecular formulae (richness), **B)** Pielou's evenness, **C)** the Shannon index, **D)** functional diversity of mass, **E)** functional diversity of number of N atoms, and **F)** functional diversity of H/C ratios. Note that we added a small set-off between samples from different strains to minimize overlapping. A total of 262,143 combinations were tested for both datasets (exometabolomes and marine DOM). Circles filled black represent combinations made out of *D. shibae* exometabolomes exclusively. Circles filled white represent combinations made out of *P. inhibens* exometabolomes exclusively. Circles filled grey represent combinations of exometabolomes from both strains. The mean of exometabolomes is given as a grey line. Blue dots represent combinations from repeated measurements of the same deep water sample, its mean is given as a blue line.

**Supplementary table 1 |** Percentage of substrate left in the incubations.

|  | *D. shibae* | | | *P. inhibens* | | |
| --- | --- | --- | --- | --- | --- | --- |
| **Growth phase** | Lag | Exponential | Stationary | Lag | Exponential | Stationary |
| Glutamate | 71±4 | 20±4 | 0* | 49±3 | 9±1 | 4±1 |
| Glucose | 47±5 | 24±6 | 6±3 | 64±6 | 3±2 | 0* |
| Succinate/Acetate | 43±9 | 12±11 | 0* | 31±9 | 8±3 | 0* |

* Below detection level
